# Endocrine side effects of immune checkpoint inhibitors

**DOI:** 10.3389/fendo.2023.1157805

**Published:** 2023-05-11

**Authors:** Zulma Cardona, Jeffrey A. Sosman, Sunandana Chandra, Wenyu Huang

**Affiliations:** ^1^ Division of Endocrinology, Metabolism and Molecular Medicine, Northwestern University Feinberg School of Medicine, Chicago, IL, United States; ^2^ Division of Hematology and Oncology, Northwestern University Feinberg School of Medicine, Chicago, IL, United States

**Keywords:** immune checkpoint inhibitor (ICI), endocrine side effects, hypophysitis, thyroiditis, type1 diabetes, adrenalitis, adrenal insufficiency, central diabetes insipidus

## Abstract

Immune checkpoint inhibitors (ICIs) have increasingly been the mainstay of treatment for numerous malignancies. However, due to their association with autoimmunity, ICIs have resulted in a variety of side effects that involve multiple organs including the endocrine system. In this review article, we describe our current understanding of the autoimmune endocrinopathies as a result of the use of ICIs. We will review the epidemiology, pathophysiology, clinical presentation, diagnosis, and management of the most commonly encountered endocrinopathies, including thyroiditis, hypophysitis, Type 1 diabetes, adrenalitis, and central diabetes insipidus.

## Introduction

Since the introduction of immune checkpoint inhibitors (ICIs) to the therapeutic arsenal of oncology, their indications continue to expand and they have become the standard of care for a number of malignancies due to their efficacy based on an anti-tumor immune response and subsequent impact on overall survival (1–6). With the wide use of ICIs in cancer therapy, there has been an increased prevalence of adverse events that impact almost every organ system (7, 8). The most commonly used ICIs are the cytotoxic T-lymphocyte-associated protein 4 (CTLA-4) inhibitors (including ipilimumab and tremelimumab), programmed death 1 (PD-1) inhibitors (including nivolumab, pembrolizumab, and cemiplimab), programmed death ligand 1 (PD-L1) inhibitors (including atezolizumab, avelumab and durvalumab (9, 10)), and the lymphocyte activation gene-3 (LAG-3) inhibitor relatlimab (11). In addition, the combination of different ICIs, including nivolumab and ipilimumab or nivolumab and relatlimab has been FDA-approved for specific indications including melanoma, lung cancer, and renal cell carcinoma (11, 12).

**Figure 1:**
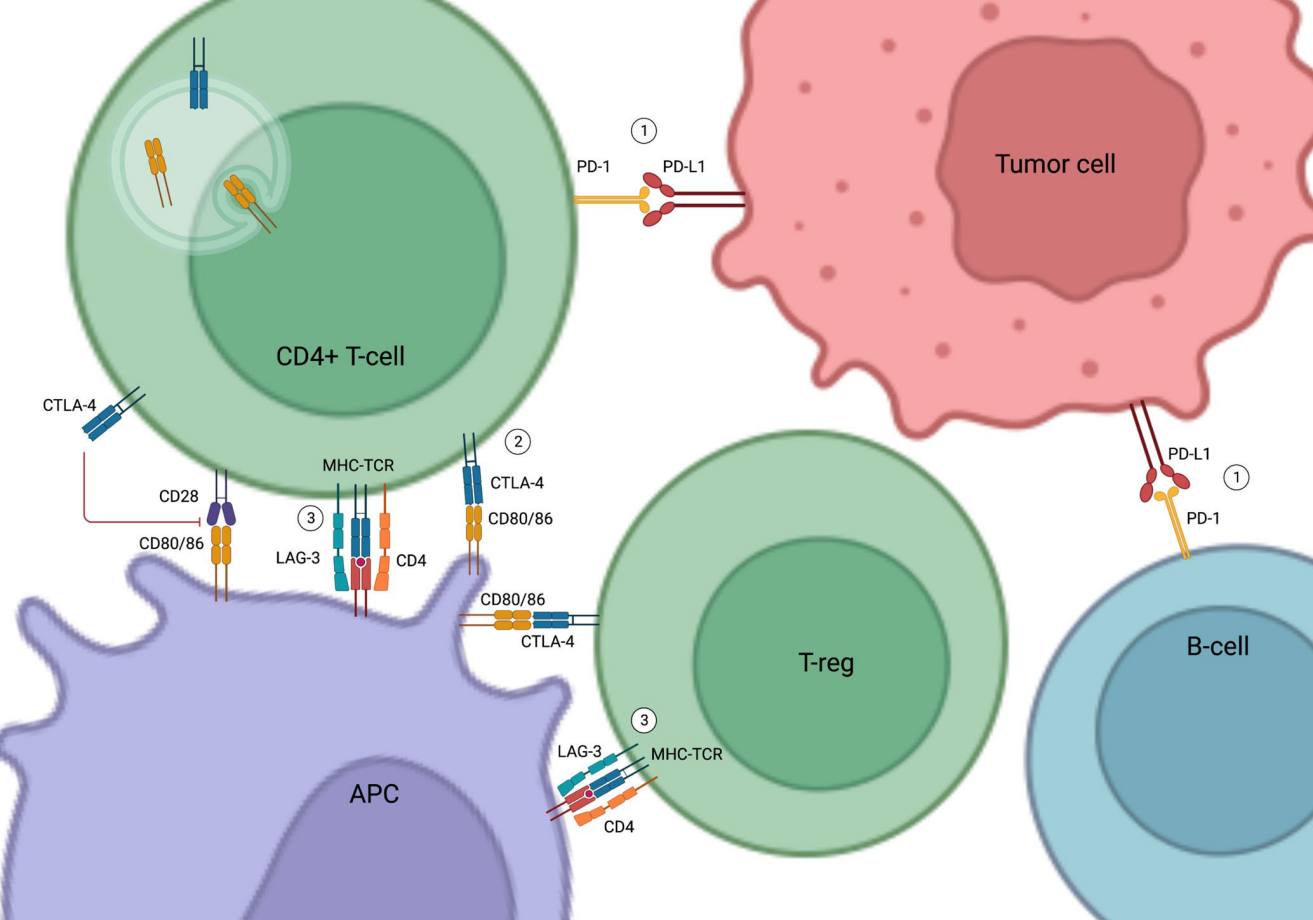
Mechanism of Action of Immune Check Point Inhibitors. (1) PD-1 and PD-L1 binding inhibits T cell activation and promotes apoptosis of tumor-infiltrating lymphocytes, resulting in the escape of the tumor cells from immune surveillance. (2) CTLA-4 inhibits T cell activity by competing with CD28 in binding to their shared ligands (CD80/CD86) on the APCs. It also initiates endocytosis of CD80/86 and leads to immune tolerance. (3) LAG-3 competes with CD4 in binding to MHC-TCR complex to inhibit T-cell activation and proliferation. LAG-3 on T-regs also suppress APCs. PD-1, Programmed cell death protein 1; PD-L1, Programmed cell death ligand 1; CTLA-4, Cytotoxic T-lymphocyte-associated antigen 4; APC, Antigen Presenting Cell; LAG-3, Lymphocyte-activation gene 3; MHC-TCR, Major histocompatibility complex-T cell receptors; T-reg, Regulatory T cells; B-cell, B lymphocytes; T-cell, T lymphocytes.

CTLA-4 is expressed on the T cell surface and regulates T cell activation and autoimmunity in association with CD28. While CD28 stimulates T cell activation and cytokine production, CTLA-4 inhibits T cell proliferation and activation by competing with CD28 in binding their shared ligands CD80 and CD86 on the antigen-presenting cells (13–16) (**Figure 1**).

PD-1 is found in various immune cells such as T cells and B cells (17). PD-L1, the natural ligand of PD-1 in tumor cells and tumor-infiltrating macrophages, binds to PD-1 to induce and maintain immune tolerance (17–19). Activation of the PD-1/PD-L1 pathway inhibits T cell activation, promotes apoptosis of tumor-infiltrating lymphocytes, decreases secretion of inflammatory cytokines, and enhances secretion of the immune inhibitory cytokine interleukin-10 (IL-10), eventually resulting in the escape of tumor cells from immune surveillance (19, 20) (**Figure 1**).

More recently, LAG-3 has emerged as a new immune checkpoint target (11, 21–24). LAG-3 is expressed on the surface of immune cells, such as T lymphocytes, and interferes with a common pathway to both CD4 and CD8 activation, thereby negatively regulating the activation of T memory cells (21, 22). LAG-3 inhibitors in combination with PD-1 inhibitors have been shown to improve the efficacy of anti-PD-1 in the treatment of advanced melanoma (11) (**Figure 1**).

T-cell activation induced by ICIs is non-specific and can trigger autoimmune reactions and lead to side effects (9, 10). The adverse events induced by ICIs are commonly referred to as immune-related adverse effects (irAEs), which can affect up to 40-50% of patients treated with ICIs (25). The most common irAEs are cutaneous adverse effects, with an estimated prevalence greater than 50% (26). Diarrhea and colitis occur in 10%-35% of patients treated with anti-CTLA-4, 1%-10% with anti-PD-1, and 15-32% with anti-PD-1 and anti-CTLA-4 combination therapy (27). Hepatitis is found in 5%-10% of individuals treated with ICI monotherapy and 25%-30% in those treated with combination therapy (28). The prevalence of rheumatic and musculoskeletal irAEs is approximately 10% in patients receiving ICI therapy (26). Other irAEs, including renal, pulmonary, neurological, cardiac and hematological AEs are less common (29–32).

Immune-related endocrine events (irEEs) are considered one of the most common irAEs, accounting for 8.1% of all cases (33–35). The thyroid is the most commonly affected endocrine organ, followed by the pituitary (36–38). Type 1 diabetes mellitus (T1DM) and primary adrenal insufficiency were reported less frequently. Fewer case reports have described hypogonadism, hypoparathyroidism, and autoimmune polyglandular syndrome associated with ICI therapy (39–47). The risk of developing irEEs varies with each specific ICI agent used as either monotherapy or in combination. A systematic review and meta-analysis compared the incidence of irEEs following ICI treatment in 39 randomized clinical trials involving more than 7,500 patients. Results showed that individuals receiving combination therapy with anti-CTLA-4 and anti-PD- had a higher incidence of thyroid dysfunction and hypophysitis when compared to patients on CTLA-4 inhibitors or PD-1 inhibitors alone. PD-1 inhibitors are associated with a higher risk of transient hyperthyroidism (often followed by hypothyroidism) as compared to PD-L1 inhibitors (9). Among all ICIs, ipilimumab was associated with the highest risk of irEEs (29.4%) including hypopituitarism (11.7%), most severe side effects (60%) and irreversible irEEs (100%). Interestingly, patients who developed irEEs had better clinical response to ICI therapy and better survival as compared to those without irEEs (48).

The exact clinical course of irEEs varies between individuals. Some irEEs occur as soon as the first infusion is administered, while others present well after discontinuing ICI therapy (1, 25). A retrospective observational study of 570 patients found that the median time to diagnosis of irEEs was 9 weeks. Patients with irEEs may present with a more indolent course or subclinical disease, which makes early recognition and diagnosis challenging (48). Additionally, the severity of these irEEs varies widely. For instance, hypothyroidism may present with a mild course, while diabetes always presents in potentially life-threatening diabetes ketoacidosis (DKA) which warrants immediate recognition and treatment (35). Additionally, diagnosis of irEEs can be challenging since subtle, non-specific symptoms at presentation may be confused as the natural course of cancer progression or other cancer therapy-associated side effects. Therefore, close monitoring of symptoms and hormonal status is critical to ensure patients’ safety.

The exact mechanism of irEEs is not completely understood, but several mechanisms have been proposed. It is suspected that ICI-induced immune activation causes the destruction of most or all the hormone-producing cells; however, there is no histological evidence to confirm this hypothesis (35). Immune-related endocrine events have a disease course similar to that of other autoimmune endocrinopathies, such as T1DM. It may start with an indolent inflammatory phase which usually evades clinical attention due to the lack of symptoms. Once symptoms start manifesting, most if not all hormone-producing cells have already been destroyed by the activated T cells. At this point, anti-inflammatory therapies such as high-dose glucocorticoids are unlikely to reverse the damage and salvage the gland’s endocrine function (49). On the contrary, other non-endocrine irAEs are thought to develop as a result of a transient inflammatory phase that may resolve with glucocorticoid therapy, ultimately restoring organ function, except for certain rheumatological manifestations and other rare toxicities that can become chronic (50, 51). As a result, irEEs are frequently irreversible.

Managing irEEs is especially challenging as they do not respond to conventional therapy used to treat other irAEs and frequently need an endocrine consultation. The standard strategy to treat other irAEs consists of holding the ICI and initiation of glucocorticoid therapy. However, as previously mentioned, irEEs generally do not improve with these therapeutic approaches and often require life-long hormone supplementation (52–54). However, distinct from non-endocrine high-grade irAEs, permanent discontinuation of ICI treatment is rarely needed.

## Thyroid

### Epidemiology

The incidence of thyroid-related adverse events can vary depending on the ICI. Thyroid dysfunction has been reported in 5% of patients receiving CTLA-4 inhibitors, 10% of individuals receiving PD-1 or PD-L1 inhibitor monotherapy, and up to 15-20% with anti-PD-1 and anti-CTLA-4 combination therapy (35). More recently, the incidence of hypothyroidism or thyroiditis was found in 18% of patients treated with anti-LAG-3 and anti-PD-1 combination therapy (11).

Hypothyroidism usually develops from destructive thyroiditis and can account for 30-40% of thyroid-irEEs with anti-PD-1 or PD-L1 therapy and up to 66% with combination therapy (35, 55–59). Most cases of thyroiditis present with hypothyroidism. In other patients, thyroiditis initially presents with a transient thyrotoxic phase that usually lasts 4-6 weeks that is usually asymptomatic, followed by hypothyroidism (60, 61). The incidence of thyroiditis may be underestimated since most clinical trials define ICI-related hypothyroidism and hyperthyroidism as separate entities instead of as different phases of a single pathology of thyroiditis (7, 35, 60). Overall, thyroiditis progresses to hypothyroidism in nearly all cases (34). The majority of ICI-induced thyrotoxicosis is caused by thyroiditis, with an incidence of 20% with anti-PD-1 therapy (7). Graves’ disease, an autoimmune thyroid disease that causes hyperthyroidism, is much less common, and the data are mostly limited to a few case reports (62–66).

### Pathophysiology

No specific risk factors, such as age or sex, have been found to predispose individuals to thyroid-related irEEs. However, some studies suggest that the presence of thyroid auto-antibodies, such as anti-thyroglobulin and anti-thyroid peroxidase, prior to starting ICI therapy may be associated with higher risk of developing thyroid-related irEEs (67–69). One prospective observational study with 209 subjects treated with PD-1 inhibitors found that the incidence of thyroid dysfunction was significantly higher in patients with positive baseline anti-thyroid antibodies when compared to those with negative antibodies (34.1% vs. 2.4%), and higher in patients with abnormal thyroid ultrasound compared to those with normal thyroid ultrasound (56.5% vs. 5.3%) (68). Another prospective observational study evaluated 66 subjects and demonstrated that 75% of patients who developed destructive thyroiditis had positive anti-thyroglobulin (TG) and anti–thyroid peroxidase (TPO) at baseline. Only 4.8% of individuals had positive baseline antibodies in the group that did not develop thyroiditis. Thus, positive TG and TPO antibodies at baseline are associated with a higher risk of developing destructive thyroiditis due to immune checkpoint inhibitor therapy (67).

Increased cytokine levels detected post-PD-1 inhibitor exposure may also be associated with post-ICI thyroid dysfunction (70). A recent case-control study of 26 patients assessed thyroid function, autoantibodies, cytokines, and chemokines at baseline and after the first ICI administration. Higher levels of Interleukin IL-1β, IL-2, and granulocyte-macrophage colony-stimulating factor (GM-CSF) at baseline, an early increase in serum thyroglobulin and thyroid autoantibodies, and an early decrease of IL-8, G-CSF, and MCP-1 were found in the ICI-induced thyroid disease group (70). However, thyroid transcription factor-1 (TTF-1) expression has not been associated with ICI-related thyroid events (71). Collectively, these studies suggest that the presence of anti-thyroid antibodies at baseline or their early increase after treatment or the expression of specific cytokines/chemokines may be useful to identify individuals at higher risk of developing thyroid dysfunction. However, it is worth noting that no study has demonstrated that patients with prior autoimmune thyroid disease are predisposed to experiencing more severe toxicities (34).

Interestingly, the development of thyroid-related events has been associated with improved survival and cancer-specific outcomes, the underlying mechanism of which is not well understood (72). One study involving 1,781 patients discovered that among the group with normal baseline TSH, those who developed ICI-related thyroid dysfunction had improved overall survival (median 41 months), compared to those who did not have thyroid adverse effects (median 22 months). Although abnormal baseline TSH was associated with worse overall survival, initiation of levothyroxine after ICI therapy in those with abnormal TSH was associated with improved overall survival (73).

### Presentation

The median time to develop ICI-related thyroid is approximately 6 weeks after ICI initiation. However, it can happen at any time during therapy. Patients who develop ICI-related hypothyroidism, may remain asymptomatic and can be detected during routine lab monitoring, or it can present with typical hypothyroid symptoms such as fatigue, weight gain, depression, constipation, and altered mental status when it’s very severe. Those who first present in the thyrotoxic phase of thyroiditis may experience symptoms of thyrotoxicosis, including weight loss, anxiety, palpitations, and tremors. Severe thyrotoxicosis such as thyroid storm is quite rare, but has been reported (45, 74, 75). Development of post-ICI Grave’s disease has also been described in a few case reports (62–66). There have been no reports of toxic multinodular goiters or toxic autonomous nodules being induced or exacerbated by ICI therapy.

TSH level is the preferred and most sensitive biochemical test to diagnose thyroid dysfunction in patients treated with ICI. An elevated TSH is expected to be seen in primary hypothyroidism due to thyroiditis. It’s not recommended to check anti-thyroid antibodies once hypothyroidism is diagnosed since these antibodies are generally positive in over 10% of the general population with normal thyroid function (76–78). Additionally, the presence of antibodies would not alter the clinical management of hypothyroidism (79). Distinct from primary hypothyroidism, central hypothyroidism is caused by pituitary disorder and is characterized by low free T4 with low normal or suppressed TSH, which will be discussed in detail under “Hypophysitis”.

In thyrotoxicosis, low TSH and elevated free T4 (FT4) and T3 are usually seen. However, thyroid function tests should be repeated 6 weeks after the initial diagnosis to assess for development of subsequent hypothyroidism. Individuals should be tested for Graves’ disease if the initial symptoms are severe, thyroid hormones (T4 and T3) are significantly elevated, thyrotoxic symptoms last more than 6 weeks, or other signs consistent with Graves’ disease are present including orbitopathy or a large goiter. Diagnosis of Graves’ disease is made by a positive thyroid stimulating immunoglobulin and/or thyrotropin receptor antibodies, or an increased and diffuse radioactive iodine uptake by the thyroid (80).

### Diagnosis

All patients treated with ICIs should have their thyroid function assessed every 4-8 weeks or more frequently if clinically indicated (81, 82). Some physicians monitor thyroid function at every ICI cycle (35). If TSH is elevated more than 10 mIU/mL on two separate occasions or if TSH is mildly elevated with low free T4 level, the patient should be diagnosed with primary hypothyroidism. No further testing is required, and thyroid hormone replacement should be initiated. If TSH is elevated, but less than 10 mIU/mL with a normal free T4 level, then a diagnosis of subclinical hypothyroidism is made. In this case, treatment should be individualized according to each patient’s symptoms, age, and comorbidities (78).

When a low TSH is found with elevated free T4 and/or T3, a diagnosis of thyrotoxicosis is suggested. However, if both low TSH and low free T4 are found, then a central hypothyroidism should be diagnosed and patients should be considered for thyroid hormone replacement accordingly (78).

### Management

Treatment of ICI-induced hypothyroidism is similar to the that of primary hypothyroidism and consists of thyroid hormone replacement usually in the form of levothyroxine. If the patient has significant comorbidities and is more advanced in age, then a lower starting dose of levothyroxine, such as 25-50 mcg daily should be recommended (78). TSH levels should be monitored in 6-8 weeks after treatment initiation to allow adequate time for TSH to reach stable level. In most patients with primary hypothyroidism, levothyroxine dose should be titrated to achieve a normal TSH level. However, in the case of central hypothyroidism, free T4 instead of TSH should be monitored for thyroid hormone replacement, as TSH is not reliable due to pituitary damage. Importantly, if a diagnosis of central hypothyroidism is made, or there is a concern for concomitant adrenal insufficiency, cortisol levels should be assessed and treated before starting thyroid hormone to avoid precipitation of an adrenal crisis (78).

Several studies have demonstrated that patients with thyroid-related irEEs do not particularly benefit from treatment with high-dose glucocorticoids. Thus, it is not recommended to treat ICI-induced thyroid disease with high-dose glucocorticoid therapy (60, 83–85). One study of 53 patients with ICI-related thyroid disorders showed that high-dose glucocorticoid treatment did not result in differences in the median duration of thyrotoxicosis, median time to develop hypothyroidism, median time to onset of hypothyroidism and median maintenance dose of levothyroxine (85).

Treatment of ICI-induced thyrotoxicosis due to destructive thyroiditis is mostly supportive, since the thyrotoxic phase is usually transient. Patients can be treated with beta-blockers to alleviate symptoms. If ICI-induced Graves’ disease is diagnosed, anti-thyroidal medications such as methimazole, radioactive iodine, or surgery can be considered depending on the patient’s preference and specific clinical presentation (80).

## Pituitary

### Hypophysitis

#### Epidemiology

Hypophysitis is defined as inflammation of the pituitary gland and infundibulum, commonly due to infectious, infiltrative, neoplastic, or autoimmune processes (86). In general, autoimmune hypophysitis is considered a rare condition with the true incidence unknown. Some reports estimate an incidence of 1 in 9 million individuals (87). Nonetheless, immune-related hypophysitis is the most commonly induced by the CTLA-4 inhibitor ipilimumab, with an incidence from 1.8% to 17% (88). Hypophysitis has also been reported in patients receiving anti-PD-1/PD-L1 therapy, although much less frequent, with an incidence of 0.5-1% (35, 89, 90). Additionally, those receiving combination therapy with anti-CTLA-4 and anti-PD-1 were more likely to develop hypophysitis compared to anti-CTLA-4 monotherapy (9). In a recent study, combination of LAG-3 and PD-1 inhibitors appears to have a higher incidence of hypophysitis when compared to PD-1 inhibitor monotherapy (2.5% vs. 0.8%) (11, 91).

Multiple endocrine axes can be affected by hypophysitis which leads to secondary adrenal insufficiency, central hypothyroidism, hypogonadotropic hypogonadism, or much less commonly central diabetes insipidus (CDI). A retrospective study analyzed 49 patients with melanoma treated with ipilimumab and found that 34 out of 49 patients had low testosterone levels at some point during their treatment, and 4 patients developed hypophysitis which eventually progressed to hypopituitarism (92). CDI is rare and is caused by deficiency of the antidiuretic hormone (ADH). CDI has been reported as an isolated ICI-endocrinopathy or in the context of hypophysitis (93–102). Interestingly, one recent study found that the incidence of CDI was 4% in patients with ICI-induced hypophysitis, compared to 38% in those with primary hypophysitis, highlighting the difference between these two types of hypophysitis (103).

No concrete risk factors have been identified for development of hypophysitis, but a small series suggested that age and male sex are potential risk factors. In 154 patients with melanoma treated with ipilimumab, 11% developed hypophysitis in which male gender and older age were identified as risk factors (89). A recent case-control study found that in 22 patients who developed hypophysitis, the presence of anti-pituitary antibodies and specific HLA alleles were associated with ICI-hypophysitis (104).

#### Pathophysiology

The pathogenesis of anti-CTLA-4-induced hypophysitis remains unknown. However, recent studies suggest a possible type II hypersensitivity autoimmune reaction mediated by IgG or IgM antibodies, along with the presence of anti-thyrotroph, anti-corticotroph, and anti-gonadotroph antibodies are involved in the development of anti-CTLA-4-induced hypophysitis (9). There is also evidence of a possible concomitant Type IV sensitivity reaction (T cell-mediated) contributing to inflammation. Additionally, CTLA-4 is expressed in the pituitary glands of C57BL/6 mice, which may explain why hypophysitis is more common in those treated with anti-CTLA-4 therapy (9, 35, 105).

#### Presentation

Most patients with hypophysitis are symptomatic. Studies suggest that the median time to presentation is 9-12 weeks in patients treated with anti-CTLA-4 monotherapy or combination immunotherapy, while the median time to presentation in patients treated with anti-PD-1/L1 therapy is 26 weeks (89).

Symptoms are related to specific hormone deficiencies such as adrenal insufficiency, central hypothyroidism, hypogonadism, and diabetes insipidus. Patients with adrenal insufficiency may experience fatigue, dizziness, nausea, vomiting and weight loss. Hypogonadal symptoms in men include fatigue, loss of muscle mass, decreased libido, and erectile dysfunction (106, 107). However, some of these symptoms might be experienced by patients undergoing ICI therapy regardless of testosterone levels. Concomitant hypogonadism may exacerbate these symptoms and negatively impact quality of life (106, 107). Women with hypogonadism may present with oligomenorrhea or amenorrhea. Patients with CDI may manifest with polyuria, polydipsia, extreme thirst, and excessive nocturia. Of note, other symptoms, such as headaches, visual changes, nausea, and emesis, can be seen in some patients and are due to cerebral edema and mass effect (34).

#### Diagnosis

Biochemical evaluation should be performed when there is clinical suspicion for hypophysitis based on symptoms. An 8AM cortisol concentration of < 3 μg/dL with a low to inappropriately normal ACTH level are diagnostic for secondary adrenal insufficiency. If the morning cortisol is mildly low to low-normal (3–10 μg/dL), then a cosyntropin stimulation test should be considered for the diagnosis of adrenal insufficiency. Importantly, if a patient has been exposed to glucocorticoids chronically (>2 weeks of continuous use of prednisone 7.5m daily or equivalent), iatrogenic adrenal insufficiency due to exogenous glucocorticoid can develop. In such cases, an endocrinology consult is recommended (108, 109) to assist in evaluating whether the adrenal insufficiency is due to hypophysitis or exogenous glucocorticoid use is critical as the former one is usually permanent and irreversible while the latter is commonly temporary.

As previously discussed, central hypothyroidism is characterized by low free T4 and low or low normal TSH. Hypogonadotropic hypogonadism is assessed by measuring early morning testosterone or estrogen, LH, and FSH after a 12-hour fast. Results consistent with hypogonadotropic hypogonadism include low morning testosterone or estrogen, with a low or inappropriately normal LH and FSH (35). However, it is essential to keep in mind that diagnosing hypogonadism in this patient population can be quite challenging as they can typically experience transient hypogonadism during acute stress or illness, which includes undergoing cancer treatments. And this transient process would not necessarily be considered true hypogonadism.

When CDI is suspected due to polydipsia, polyuria, nocturia, and extreme thirst, the first step is to differentiate it from primary polydipsia and nephrogenic diabetes insipidus (DI) (42, 95, 97–99). Hypernatremia in the setting of dilute urine confirms the diagnosis of diabetes insipidus and rules out primary polydipsia. However, patients often present with normal serum sodium levels due to their intact thirst and excessive water consumption. A water deprivation test or a hypertonic saline infusion test with serum copeptin measurements may help confirm the diagnosis of DI. The differentiation between CDI and nephrogenic DI can be made when exogenous ADH (desmopressin) is given after water deprivation or hypertonic saline infusions. A positive response to desmopressin is considered for the diagnosis of CDI when extreme thirst, fluid intake and urine output significantly decrease and urine osmolarity increases. While in nephrogenic DI, such clinical and biochemical responses are lacking. Copeptin is a peptide derived from the C-terminus of the pre-pro-hormone of ADH and is a more reliable marker for ADH release than the direct measurement of ADH level. Copeptin level is usually low in CDI, especially after water deprivation or a hypertonic saline challenge (110, 111). Once CDI is diagnosed, an MRI of the pituitary gland can be obtained. Typically, a normal posterior pituitary shows hyperintensity on T1-weighted images, known as the “bright spot” (112). The bright spot is typically absent in CDI; however, the bright spot can be absent in up to 25% of normal individuals and can also fade with aging (112). It is important to note that normal imaging findings do not rule out hypophysitis. Patients with headaches, visual changes, or any other neurological symptoms should also have a brain MRI.

#### Management

Routine monitoring for secondary adrenal insufficiency in ICI-treated patients is controversial. Some recommend obtaining morning cortisol levels with or without ACTH at every cycle if receiving an anti-CTLA-4 inhibitor monotherapy or combination therapy, but not in those receiving anti-PD-1/PD-L1 monotherapy in the absence of symptoms. Adrenal insufficiency is treated with glucocorticoid replacement, most commonly hydrocortisone or prednisone. It is essential to educate patients about sick day dosing (doubling and tripling of daily glucocorticoid dosing during mild and moderate illness, respectively) to prevent adrenal crisis. Once the illness resolves, patients can taper to their regular replacement doses of glucocorticoids. Stress dosing is needed when the patient is severely sick or is undergoing a procedure/surgery. In these cases, hospitalization for IV hydration and/or parenteral glucocorticoid therapy are usually needed (34, 108). If a patient develops concomitant central hypothyroidism, they should be replaced with glucocorticoids before starting thyroid hormone to avoid adrenal crisis (108). Higher doses of glucocorticoids could be indicated in the treatment of patients experiencing hypophysitis-related severe compressive symptoms such as headache and vision change. Some recommendations include therapy with prednisone 1-2 mg/kg with a taper over 1-2 weeks (82, 84).

There are conflicting results regarding the effect of glucocorticoid therapy on tumor response to ICI therapy and overall survival (89, 113–115). At present, it is generally recommended to reserve high-dose glucocorticoids to treat severe symptoms such as headaches which could be due to pituitary gland and sella turcica enlargement, and severe hyponatremia. Similar to ICI-related thyroid disorders, immunotherapy does not have to be discontinued once patients develop hypophysitis as long as they are receiving appropriate hormone replacement therapy. However, it would be reasonable to pause ICI therapy until the resolution of severe symptoms (35).

For male hypogonadotropic hypogonadism, testosterone replacement can be started if indicated and there is no contraindication (35).

If a patient is diagnosed with ICI-CDI and has an intact thirst mechanism and free access to water, then desmopressin can be initiated and titrated to control extreme thirst and polyuria (95). If the patient develops severe dehydration and hypernatremia, it will be considered a grade 4 ICI toxicity and ICI therapy should be temporarily suspended (84, 116, 117). Suspending ICI therapy would allow time for desmopressin titration to safely correct hypernatremia and water deficit. Once the patient improves and is stable, ICI therapy can be resumed (84, 116, 117).

## Adrenal

### Primary adrenal insufficiency

#### Epidemiology

The development of ICI-induced primary adrenal insufficiency (ICI-PAI) is rare, with only a few cases reported in the literature (9, 47, 118, 119). The incidence of ICI-PAI is 1.03% in all reported cases of irAEs and between 0.60 and 0.77% in individuals on monotherapy. Combination therapy with CTLA-4 and PD-1 inhibitors was associated with a higher risk of PAI compared with PD-1 inhibitor monotherapy (120). It’s also been reported that male and elderly individuals have a higher risk of ICI-PAI. Additionally, lower-weight in this patient population was associated with poor clinical outcomes (120).

#### Pathophysiology

ICI-PAI develops as a result of T-cell and antibody-mediated destruction of the adrenal cortex. A recent study showed uniformly increased uptake of 18F-fluorodeoxyglucose in the adrenal glands in patients with ICI-PAIs, suggesting that an inflammatory process may be the underlying mechanism for the development of ICI-PAIs (118, 121).

#### Presentation

Patients with ICI-PAI can present with similar symptoms as those in secondary adrenal insufficiency, including fatigue, poor appetite, weight loss, nausea, and vomiting. Importantly, these patients are usually sicker and present with hypotension or adrenal crisis due to concomitant mineralocorticoid deficiency (47, 120, 122).

#### Diagnosis

PAI is diagnosed with low morning cortisol levels along with an elevated ACTH level. Electrolyte imbalances are usually present including hyponatremia (due to either glucocorticoid or mineralocorticoid deficiency), metabolic acidosis, and hyperkalemia (due to mineralocorticoid deficiency) (122). Plasma renin is usually elevated.

#### Management

PAI, if left untreated, can be life-threatening. Thus, it is important to recognize it promptly to avoid adverse consequences. Individuals with PAI should be started on glucocorticoid replacement immediately. The dose and route (oral or parenteral) should be determined depending on the patient’s clinical status. After the acute phase, the patient should remain on lifelong glucocorticoid replacement therapy. Furthermore, patients with PAI should also be started on mineralocorticoid replacement to prevent hypotension and hyperkalemia. ICI therapy does not have to be suspended or discontinued if symptoms are not severe (122, 123).

## Diabetes

### Epidemiology

Diabetes Mellitus (DM) has been reported in 0.2-1.4% of patients treated with ICI (9, 59, 124, 125). Studies showed that ICI-DM is found in 71% of individuals treated with anti-PD-1 or anti-PD-L1 monotherapy, 15% with combination therapy (PD-1/PD-L1 and CTLA-4 blockade), and 3% with anti-CTLA-4 monotherapy (7, 35, 125). Demographics reflect those typical for patients treated with ICI (100). ICI-induced DM usually presents with other irAEs, such as pancreatitis, thyroiditis, or colitis (35, 126, 127).

### Pathophysiology

The mechanism proposed for the development of ICI-induced DM is similar to that of T1DM. Presumably, ICI-DM results from the autoimmune destruction of pancreatic β-cells, which express PD-L1 (35, 128). Typically, the PD-1 and PD-L1 pathway induces T-cell apoptosis and protects pancreatic β-cells from immune cytotoxicity. PD-1 inhibition results in restoration of T cell activation and destruction of the pancreatic β-cells (128, 129).

Studies using human tissues from patients with ICI-DM are fairly limited. However, it has been reported that PD-L1 expression in human β-cells is increased in patients with T1DM when compared to health subjects (130, 131). Additionally, there is evidence of increased islet cell infiltration of CD8 T lymphocytes, similar to that observed in early-onset T1DM (130). Other studies suggest that ICI-DM happens as a result of more severe and rapid destruction of pancreatic β-cells when compared to T1DM due to an absolute absence of insulin-positive cells identified in the pancreas of patients with ICI-DM (130).

Interestingly, only 40–50% of individuals with ICI-DM have detectable islet autoantibodies at the time of diagnosis, compared with >90% in T1DM (124, 125). Patients with ICI-DM who tested positive for autoantibodies developed ICI-DM early after initiation of ICI therapy compared to individuals without these autoantibodies (124). A study of 10 patients with ICI-DM found that patients who tested negative for autoantibodies did not become autoantibody-positive until 32 months after initial diagnosis and in general, ICI-DM usually lacks antibodies typically associated with T1DM (126).

Genetic factors have also been implicated in the risks of developing ICI-DM (126). HLA haplotypes associated with T1DM (DR3-DQ2 and DR4-DQ8) and fulminant DM in Asian populations (DR4-DQ4 and DR9-DQ9) are estimated to be present in approximately 61-76% of patients with ICI-DM (124, 127). HLA-DR4 was identified as having a particularly strong association with ICI-DM development (127).

Inflammation of the pancreas induced by ICI therapy can result in exocrine dysfunction in addition to diabetes. A study reported that amylase and lipase were elevated in one-third of patients with ICI-DM and 42% had pancreatitis in the peri-diagnosis period (124). Together, all these findings suggest that the pathophysiology of ICI-DM is unique and differs from that in classic T1DM.

### Diagnosis

Screening for ICI-DM involves checking random blood glucose levels at every treatment cycle. If new onset hyperglycemia is identified, additional evaluation should include a basic metabolic panel, urinary and serum ketones, c-peptide, and hemoglobin A1c (HbA1c) (82, 132, 133). These tests will help identify patients with diabetes ketoacidosis (DKA) and distinguish between causes of hyperglycemia such as type 2 diabetes mellitus (T2DM), steroid-induced hyperglycemia, or stress hyperglycemia (125). A recent study showed that 59% of ICI-DM cases presented with DKA. Occasionally, concomitant pancreatitis is also present (124).

### Management

It is estimated that approximately 60-85% of cases of ICI-DM present in DKA (134). Patients with DKA should receive immediate treatment according to standard DKA protocol, which includes continuous intravenous insulin infusions, fluid resuscitation, and correction of electrolyte abnormalities (135). Patients will need insulin therapy permanently, and management should be similar to that of classic T1DM (135). As mentioned previously for other irEEs, high-dose glucocorticoids are not recommended as they have not been shown to improve the clinical course or outcome of ICI-DM. More importantly, high-dose glucocorticoids can exacerbate hyperglycemia in patients with diabetes. One study suggested that mesenchymal stem cell (MSC) therapy may prevent development of ICI-DM in a NOD mouse model. In this study, MSCs were found to decrease the amount of T-cells and CXCL9-positive macrophages in the islet cells and the occurrence of diabetes. The insulin content and islet beta cell area also improved (1.9-fold and 2.7-fold, respectively). However, additional studies are required in order to prove this novel approach (136).

## Hypoparathyroidism

ICI-induced hypoparathyroidism is an extremely rare endocrine side effect that has only been reported in 4 case reports so far (137–140).

### Pathophysiology

The mechanism of ICI-induced hypoparathyroidism remains unclear. Proposed mechanisms include cell damage induced by an autoantibody against the parathyroid glands and inhibition of parathyroid hormone secretion by activating calcium-sensing receptor autoantibodies (137, 140, 141).

### Presentation

Patients may present with mild hypocalcemia and hyperphosphatemia. Symptoms of hypocalcemia include perioral tingling and numbness, muscle spasms and cramping, and even tetany (140). Severe life-threatening hypocalcemia can also be seen which would require prompt recognition to prevent poor outcomes.

### Diagnosis

Patients should be evaluated with a renal panel that includes albumin and phosphorus levels (140). Calcium level should be corrected for the albumin level in case of hypoalbuminemia. Once hypocalcemia is confirmed, PTH levels should be measured. A diagnosis of hypoparathyroidism is confirmed if PTH level is low to inappropriately normal in the setting of hypocalcemia (142).

### Management

The main focus of managing ICI-induced hypoparathyroidism is maintaining normal calcium levels *via* calcium, Vitamin D, and sometimes calcitriol supplementation. Hypercalciuria should be monitored in these patients when their calcium level improves. In severe cases, PTH analogs can be used (142). ICI-induced hypoparathyroidism, like other irEEs, is believed to be permanent (140).

## Conclusion

ICI therapy has revolutionized cancer treatment over the past decade. As their use expands in the adjuvant and metastatic settings in numerous cancers, an increase in the spectrum of irAEs will likely be observed. The previously recognized rare irAEs may be more frequently encountered, requiring more attention from the treatment team. Ideally, these cases should be addressed in a multidisciplinary approach, including oncology and endocrinology working together to aid in the management of these irEEs.

There are several challenges that need to be addressed regarding the diagnosis and management of irEEs. One of the challenges is to identify potential risk factors for the irEEs, as this knowledge could possibly impact the process of ICI therapy selection, tailor specific clinical monitoring, and may help to identify appropriate populations for specific screening approaches. Additionally, irEEs could potentially represent an exceptional opportunity to explore the mechanisms underlying endocrinopathies within and outside the context of ICI therapy. Additionally, irEEs may be a surrogate for the investigation of the pathophysiology of other irAEs due to the ease of acquisition of certain endocrine tissues (35). The thyroid gland, for example, may be great for sampling and studying the inflammatory process to better elucidate mechanisms behind irAEs due to its ease of access and the high incidence of thyroiditis seen with ICI therapy. It would also be interesting to further investigate the associations between thyroid dysfunction, hypophysitis, and improved cancer-specific survival, which may provide insights into the interplay among the immune system, cancer tumor microenvironment, and endocrine organs (72).

Larger studies are needed to further investigate the safety of glucocorticoid therapy and its potential effects on the anti-tumor immune response and survival outcomes. Ultimately, understanding the side effects associated with immunotherapy will allow for prompt recognition and facilitate earlier treatment delivery to prevent adverse outcomes from irEEs.

## Author contributions

All authors contributed to the manuscript. Specifically, ZC and WH wrote the initial draft. JS and SC made significant revisions. All authors contributed to the article and approved the submitted version.
